# Alexithymia and metabolic syndrome: the mediating role of binge eating

**DOI:** 10.1007/s40519-020-00964-x

**Published:** 2020-09-13

**Authors:** Chiara Conti, Giulia Di Francesco, Melania Severo, Roberta Lanzara, Katie Richards, Maria Teresa Guagnano, Piero Porcelli

**Affiliations:** 1 Department of Psychological, Health, and Territorial Sciences, University “G. d’Annunzio” of Chieti‐Pescara, Via dei Vestini 31, 66100 Chieti, Italy; 2 Department of Dynamic and Clinical Psychology, “Sapienza” University of Rome, Rome, Italy; 3 Department of Psychological Medicine, King’s College, London, UK; 4 Department of Medicine and Aging, University “G. d’Annunzio” Chieti‐Pescara, Chieti, Italy

**Keywords:** Alexithymia, Binge eating, Distress, Metabolic syndrome, Obesity, Structural equation modeling

## Abstract

**Purpose:**

Alexithymia, a personality trait characterized by difficulties in emotional processing, has been associated with unhealthy behaviors and chronic medical conditions. This study aimed to further develop our understanding of this complex relationship by investigating whether alexithymia increases the risk of metabolic syndrome (MetS) in participants with obesity or overweight through the mediating role of binge eating (BE).

**Methods:**

A consecutive sample of 238 treatment-seeking patients with obesity or overweight were recruited. Alexithymia (TAS-20), binge eating symptoms (BES), body mass index (BMI), and depression and anxiety symptoms (HADS) were concurrently assessed.

**Results:**

Almost half of the participants met the criteria for MetS (44.12%). Compared to patients without MetS, those with MetS were older, had a longer duration of overweight, and had a higher BMI (*p* < 0.01). Individual with MetS also had higher HADS, BES, and TAS-20 scores, particularly difficulty identifying and describing feelings. The structural equation modeling (SEM) analysis revealed that BES levels exerted a significant direct effect on MetS (*p* < 0.01), and that TAS-20 levels exerted a significant direct effect on BES (*p* < 0.01), anxiety (*p* < 0.001) and depression (*p* < 0.001). Moreover, psychological distress (anxiety, *p* = 0.01, and depression, *p* = .05) indirectly affected MetS through the mediating effect of BES, and TAS-20 (*p* = 0.01) indirectly affected MetS through the mediating effect of HADS and BES. Finally, age had a significant direct effect on MetS (*p* < 0.001).

**Conclusion:**

Our findings indicate that alexithymia is a concurrent causative factor to the development of MetS through the mediating role of BE and psychological distress.

**Level of evidence:**

Level III, case–control analytic study.

## Introduction

Globally, both obesity and metabolic syndrome (MetS) are growing public health problems [1]. The prevalence of both conditions has increased dramatically in industrialized countries over the past 30 years [2]. The prevalence of obesity doubled from 1975 (34 million men and 71 million women) to 2014 (266 million men and 375 million women) [3]. MetS is more common among individuals with obesity than normal weight individuals, with approximately 50% of women with obesity and nearly 60% of men with obesity experiencing MetS [4].

MetS is a combination of clinical criteria including abdominal obesity, metabolic abnormalities (high levels of glycaemia and blood pressure), and altered lipid metabolism (high levels of triglycerides and cholesterol) (see Methods 2.2.2 below), and is considered a major risk factor for cardiovascular disease [5]. MetS is highly prevalent among adults, ranging 7–34% in men and 5–22% in women [6], but can also affect children and adolescents. Its prevalence increases with age, with up to 44% of adults aged 40 and older presenting with MetS compared to 7% of younger adults aged 20–29 [7]. Furthermore, psychological distress is thought to increase the risk of MetS. Subjects with high psychological distress at baseline are more than twice as likely to develop MetS than those with low psychological distress. Pooled finding from cross-sectional studies show that anxiety and major depression are associated with an elevated risk of MetS (OR = 1.07, 95% CI 1.01–1.12 and OR = 1.82; 95% CI  = 1.06–3.14, respectively) [8, 9]. Several of the identified causes and risk factors for obesity and MetS are related to unhealthy lifestyles, such as poor dietary patterns, sedentary lifestyle, and decreases or increases in sleep duration [10].

Binge eating (BE) is one behavior that has been identified as clinically relevant for the development of combined obesity and MetS [11–13]. BE involves the consumption of an unusually large amount of food within a discrete time period alongside a perceived inability to stop or control eating [14]. Recurrent BE episodes, at least once a week for three months, are a hallmark feature of binge eating disorder (BED). Other key features of BED include a lack of compensatory behaviors such as excessive exercise or vomiting, feeling depressed, embarrassed and disgusted with oneself after BE, and experiencing substantial distress regarding binge episodes [14]. BED has been associated with the severity of obesity [12], and general psychopathology, such as anger and impulsivity [15]. BED is typically managed with psychological and pharmacological interventions [16]. Compared with non-BE individuals with obesity, binge eaters with obesity engage in maladaptive eating behaviors and patterns that may place them at greater risk for MetS [17]. For example, hypercaloric food intake has been linked with more non-alcoholic fatty liver disease and elevated lipids in men and women with severe obesity [18]. A longitudinal study demonstrated that binge eaters were at greater risk for newly diagnosed MetS components, compared with non-binge eaters [11]. Despite the evidence for the co-occurrence of obesity with BE and MetS, little is known about the psychological characteristics of patients with obesity and MetS. In particular, it is still uninvestigated whether and how psychological characteristics are associated with different developmental trajectories of dieting, BE, and obesity problems.

Alexithymia is a personality dimension that has been associated with an increased incidence of numerous physical illnesses. The association between alexithymia and physical ill health is complex, alexithymia may modulate illness severity, pre-dispose an individual, be a consequence of the illness, or may involve a complex combination of all of these roles [19]. It is a multifaceted personality dimension composed of two higher‐order factors: a deficit of affect awareness [as difficulty identifying feelings [DIF] and difficulty describing feelings (DDF)] and operatory thinking (externally oriented thinking [EOT] and poor imaginal processes) [19]. These characteristics are thought to reflect deficits in the cognitive processing and regulation of emotions associated with anxiety and depression [20], and can affect health-related behaviors [21], such as dieting habits and physical activity [22]. Not surprisingly, alexithymia has been related to the clinical manifestation and maintenance of obesity [23] and a range of maladaptive eating patterns, including BE [24, 25]. Eating behaviors are known to be highly influenced by emotional regulation, providing a strong rationale for considering alexithymia as a potential predictor of obesity and BE [26]. Several neuroimaging studies have revealed that alexithymic traits are associated with activation in brain regions that subserve interoception and physiological monitoring [27, 28]. This is interesting in light of recent developments in the treatment of obesity. There is a growing literature demonstrating that mindfulness-based interventions, many of which stress increased attention to interoceptive information from the body, are an effective tool for promoting healthier eating behaviors [29].

Previously published studies mainly focused on the association of alexithymic features with single risk factors or components of MetS. The severity of alexithymia may contribute indirectly to MetS by influencing some of its components, such as T2D and cardiovascular risk [29, 30]. Given that alexithymia is thought to reflect deficits in mentalizing emotions, individuals with high levels of alexithymia may be more prone to experiencing psychological distress, anxiety, and depression [31–34]. Therefore, the relationship between alexithymia and MetS could be mediated through both BE and psychological distress. Moreover, to our knowledge, BE has not been considered as a co-variable in previous studies exploring the relationship between alexithymia and MetS. In this study, we aimed to explore the association between alexithymia and MetS by including maladaptive eating behavior and psychological distress as mediating factors. In particular, the aims of this study are: (a) to investigate whether patients with MetS have higher alexithymic traits, BE, depression, and anxiety than those without MetS, (b) to investigate whether patients with both MetS and BE have higher alexithymic traits than all other patients; and (c) to explore whether and to what extent psychological features (i.e., alexithymia, BE, depression, and anxiety) are associated with MetS. Based upon the previous literature, we expected that: (a) patients with MetS would exhibit more features of alexithymia, BE, depression, and anxiety than patients without MetS; (b) patients with both MetS and BE would have higher alexithymic traits than all other patients; and (c) psychological features (i.e., alexithymia, BE, depression, and anxiety) would be associated with MetS, and in particular alexithymia would indirectly affect MetS through the mediating role of BE, and depression and anxiety symptoms rather than directly.

## Materials and methods

### Participants

A sample of 297 treatment-seeking outpatients with obesity or overweight were consecutively enrolled at their first visit at the Obesity Centre at the University Clinical Hospital of Chieti (Italy). At intake subjects were first screened for medical conditions and their motivation to participate in the weight loss program. The weight loss program was characterized as an integrated approach including daily food intake monitoring, the promotion of a healthy lifestyle, behavioral modification of risk factors associated with eating behavior, and psychological counseling aimed at individual and interpersonal distress. To maximize the ecological validity of the sample, all adult outpatients from 18 to 70 years old with a Body Mass Index (BMI) ≥ 25 were invited to take part. Individuals were excluded if they self-reported a psychiatric or neurological disorder or the use of psychoactive substances in the past 10 years, were pregnant, were unable to complete the self-rating scales, or had documented or self-reported type 1 diabetes or any other endocrinological problem other than MetS.

All subjects provided written informed consent to take part in the study. The study was reviewed and approved by the Ethics Committee of University G.d'Annunzio—Chieti-Pescara and carried out in accordance with the World Medical Association Declaration of Helsinki and its subsequent revisions [35].

### Measures

#### Socio-demographic and clinical characteristics

An ad hoc semi-structured interview was used to gather information on socio-demographic and clinical characteristics, such as age, gender, BMI, education level, marital status, and duration of overweight (years from the first dietary treatment), past and/or current psychopathological and neurologic symptoms, use of psychiatric medication, and experiences with psychotherapy. A BMI ≥ 30 was used as the cut-off for determining obesity and was analyzed as the ratio of weight in kilograms to the square of height in meters (kg/m2).

#### Clinical definition of MetS

In the present study, MetS was clinically defined using the NCEP–ATP III diagnostic criteria [36].

According to the polythetic NCEP–ATP III criteria [36], MetS is diagnosed when at least three of the following are satisfied: central obesity (defined as waist circumference ≥ 88 cm in females, and ≥ 102cmin males); triglycerides of 150 mg/dL or greater (1.7 mmol/L) or specific treatment for hypertriglyceridemia; HDL cholesterol less than 40 mg/dL (1.03 mmol/L) in males, and less than 50 mg/dL (1.29 mmol/L) in females or specific treatment for hypercholesterolemia; systolic blood pressure of 130 mm Hg or greater and/or diastolic blood pressure of ≥ 85 mm Hg or treatment for previously diagnosed hypertension; FPG of 100 mg/dL or greater (5.6 mmol/L) or previously diagnosed type 2 diabetes.

#### Binge eating (BE)

The Binge Eating Scale (BES) was used to assess the severity of BE behaviors [37]. The BES is a 16-item self-report questionnaire with scores ranging from 0 (no BE) to 46 (severe BE). Scores of ≥ 27 have typically served as the cut-off value for detecting the presence of severe BE, ≥ 18 for moderate BE, and ≤ 17 for minimal or no BE. For our sample, Cronbach’s α was 0.88.

#### Alexithymia

The self-report 20-item Toronto Alexithymia Scale (TAS-20) was used to measure Alexithymia [38]. Each item is rated on a 5-point Likert scale, with total scores varying from 20 to 100. The TAS-20 consists of three subscales: the DIF, the DDF, and the EOT. The DIF subscale measures an individual’s capacity to identify feelings and discriminate them from bodily sensations. The DDF subscale evaluates an individual’s capacity to describe feelings to other people. The EOT subscale assesses the propensity of an individual to orientate their attention towards external over internal events. A score 61 or above is typically used as the cut-off for high alexithymic traits [39]. The scale is considered the standard measure for alexithymia because of its good psychometric properties including internal consistency, construct validity, and factor structure that have been demonstrated across the world [40]. For our sample, Cronbach's α was 0.83 for the total scale, 0.85 for the DIF, 0.70 for the DDF, and 0.56 for the EOT subscales.

#### Depressive and anxiety symptoms

Depressive and anxiety symptoms were measured using the Hospital Anxiety and Depression Scale (HADS) [41]. The HADS is a 14-item self-report scale that is broadly used to assess psychological distress in patients with medical health problems and contains two distinct 7-item subscales for depression (HADS-D) and anxiety (HADS-A). Items on each subscale are rated on 4-point Likert scales ranging from 0 (no symptom) to 3 (definite experience of symptoms). For each subscale, scores between 8 and 10 are classed as borderline cases and scores from 11 to 21 are considered as moderate to severe for symptoms of anxiety and depression. The HADS has been used in several medical settings, indicating good reliability and validity [42]. For our sample, Cronbach’s α for HADS-D was 0.78 and for HADS-A 0.81.

### Statistical analysis

A 2-step strategy was used for data analysis.

First, Student’s *t* test or chi-square test (*χ*^2^) and an analysis of variance (ANOVA) were used to compare between-group differences in socio-demographic and clinical variables for patients with or without MetS and BE. The standardized mean difference was used as a measure of effect size. A standardized effect size (Cohen’s *d*) of 0.20–0.50 is considered small, 0.50–0.80 moderate, and > 0.80 large [43]. The Eta-squared (*η*^*2*^), a measure of effect size for ANOVA, was also used. A standardized effect size (*η*^*2*^) of 0.01–0.05 is considered small, 0.06–0.14 moderate, and > 0.14 large. Pearson’s correlation coefficient was used for the associations between socio-demographic, clinical and psychological variables.

Second, Structural Equation Modeling (SEM) was used to evaluate the direct, indirect and total effects of alexithymia on MetS through the mediating role of BE, depression and anxiety symptoms. SEM is a set of statistical techniques used to measure and analyze the relationships of observed and latent variables. It examines linear causal relationships among variables, while simultaneously accounting for measurement error. Latent variables or factors are considered to represent theoretical constructs that can be interpreted as latent traits or “true” variables underlying the measured items. Observable variables or factors are variables that can be observed and directly measured [44].

SEM, with a maximum likelihood estimation method, was used to evaluate the fit of the hypothesized model based on the following criteria: chi-squared (*χ*^2^) (*p* > 0.05), Root Mean Square Error of Approximation (RMSEA) close to 0.06 or less for a well-fitted model, Comparative Fit Index (CFI) near 0.90 or greater and Tucker–Lewis Index (TLI) near 0.90 or greater [45]. Missing data were replaced by way of multiple imputation algorithms. The structural components of the model included one exogenous latent trait (alexithymia), one endogenous observed factor (MetS), one latent mediated variable (BE), two continuous mediator variables (depression and anxiety symptoms), and one observed covariate (age). Hypotheses regarding the structural relationships among the constructs in the final model were evaluated using the magnitude of path coefficients (standardized coefficient) and their significance.

## Results

### Characteristics of the sample

Of the 297 recruited patients, 238 (80.1%) were enrolled. The main reason for not participating was a lack of time. Included patients were mostly females (69.33%) and with a median age of 48 years. Most of the participants had graduated from high or secondary school (73.95%) and were married (74.14%). According to the polythetic NCEP–ATPIII criteria (see Methods 2.2.2), MetS was present in 44.12% of the sample.

### Between-group comparisons

#### MetS

Compared with No-MetS, patients with MetS had a higher BMI (*d* = 0.39), duration of overweight (*d* = 0.68), and older age (*d* = 0.74). No other sociodemographic differences were found between the two groups. BE behaviors, alexithymia, and psychological distress showed significant between-group differences, with effect sizes in the small range. In particular, patients with MetS reported significantly higher BES (*d* = 0.26), TAS-20 (*d* = 0.43), DIF (*d* = 0.39), and DDF (*d* = 0.34) scores than patients without MetS. Moreover, patients with MetS showed significantly higher depression and anxiety symptoms (*d* = 0.28) than patients without MetS (see Table1).

**Table 1:** Socio-demographic and clinical characteristics of the study sample (*N* = 238)

Variable	Total sample *N* = 238	MetS *N* = 105 (44.12%)	No-MetS *N* = 133 (55.88%)	*t*/*χ*^*2*^	*p*	*d*
Age (years), mean (SD)	47.39 (14.72)	53.14 (13.02)	42.84 (14.43)	5.70	< 0.001	0.74
Gender,* n* (%)						
Male	73 (30.67)	36 (34.29)	37 (27.82)	1.07	0.28	0.13
Female	165 (69.33)	69 (65.71)	96 (72.18)			
Education,* n* (%)						
Primary	12 (5.04)	8 (7.62)	4 (3)	0.81	0.41	0.10
Secondary	66 (27.73)	30 (28.57)	36 (27.07)			
High	110 (46.22)	43 (40.95)	67 (50.38)			
University	50 (21.01)	24 (22.86)	26 (19.55)			
Marital status,* n* (%)						
Unmarried	60 (25.86)	23 (22.77)	37 (28.24)	− 0.37	0.71	0.04
Currently married	172 (74.14)	78 (77.23)	94 (71.76)			
Duration of overweight (years), mean (SD)	27.94 (13.15)	32.82 (12.15)	24.24 (12.61)	4.99	< 0.001	0.68
BMI, mean (SD)	35.72 (6.97)	37.25 (7.27)	34.52 (6.49)	3.05	0.002	0.39
TAS-20 total, mean (SD)	48.94 (13.32)	52.13 (13.21)	46.41 (12.89)	3.36	0.001	0.43
DIF, mean (SD)	16.21 (7.26)	17.77 (7.44)	14.97 (6.89)	2.99	0.003	0.39
DDF, mean (SD)	13.04 (4.71)	13.94 (4.70)	12.33 (4.61)	2.65	0.008	0.34
EOT, mean (SD)	19.68 (4.94)	20.41 (4.96)	19.10 (4.85)	2.05	0.10	0.26
HADS-D, mean (SD)	6.60 (4.18)	7.26 (3.99)	6.07 (4.26)	2.20	0.01	0.28
HADS-A, mean (SD)	7.25 (4.19)	7.91 (3.78)	6.73 (4.44)	2.17	0.05	0.28
BES, mean (SD)	11.14 (7.91)	12.29 (7.65)	10.23 (8.03)	1.99	0.04	0.26

*BES* Binge Eating Scale; *BMI* Body Mass Index; *DDF* difficulty describing feelings; *DIF* difficulty identifying feelings; *EOT* externally orientated thinking; *HADS‐A* Hospital Anxiety and Depression Scale‐anxiety subscale; *HADS‐D* Hospital Anxiety and Depression Scale‐depression subscale, *MetS* Metabolic Syndrome; *No-MetS* patients without MetS; *TAS-20* 20-item Toronto Alexithymia Scale

#### MetS + BE

Results from the one-way ANOVA showed significant differences between groups, with moderate-to-large effect sizes. Patients with MetS but without BE were older (*η*^*2*^ = 0.15) than the other 3 groups and had a higher BMI (*η*^*2*^ = 0.04) and a longer duration of overweight (*η*^*2*^ = 0.13) than patients without MetS and BE. Patients without MetS and BE exhibited significantly lower TAS-20 scores than the other groups (*η*^*2*^ = 0.09), particularly lower DIF (*η*^*2*^ = 0.09). Patients with both MetS and BE had significantly higher TAS-20 (0.09) and DIF (0.09) scores than patients without MetS and BE, had significantly higher depression score (*η*^*2*^ = 0.07) than groups without BE, and significantly higher anxiety score (*η*^*2*^ = 0.07) than patients without MetS and without BE. Patients without MetS and with BE had a significantly higher TAS-20 (*η*^*2*^ = 0.09), DIF (*η*^*2*^ = 0.09), DDF (*η*^*2*^ = 0.06), and anxiety (*η*^*2*^ = 0.07) than patients without MetS and without BE (see Table 2).

**Table 2:** Socio-demographic and clinical characteristics between groups with and without MetS and BE

Variable, mean (SD)	A^a^ *N* = 30 (12.60%)	B^b^ *N* = 75 (31.51%)	C^c^ *N* = 20 (8.40%)	D^d^ *N* = 113 (47.48%)	*F*	*p*	*η*^*2*^	Games-Howell post hoc test
Age (years)	47.26 (10.88)	55.49 (13.12)	41.75 (11.39)	43.03 (14.93)	13.77	< .001	0.15	B > A; B > C; B > D
Duration of overweight (years)	28.63 (10.51)	34.53 (12.42)	29.42 (11.41)	23.30 (12.76)	11.39	< .001	0.13	B > D
BMI	37.79 (7.88)	37.03 (7.05)	36.13 (5.63)	34.23 (6.96)	3.63	.01	0.04	B > D
Alexithymia	52.73 (12.74)	51.89 (13.46)	55.35 (10.92)	44.83 (12.61)	7.78	< .001	0.09	A > D; B > D; C > D
DIF	18.66 (7.08)	17.41 (7.60)	20.15 (7.27)	14.06 (6.43)	7.69	< .001	0.09	A > D; B > D; C > D
DDF	13.96 (4.73)	13.93 (4.72)	15 (4)	11.85 (4.56)	5.05	.002	0.06	B > D; C > D
EOT	20.10 (4.03)	20.54 (5.31)	20.20 (5.05)	18.91 (4.81)	1.84	.13	0.02	–
HADS-D	9.67 (4.31)	6.33 (4.58)	8.25 (5.04)	5.70 (4.56)	5.08	.003	0.07	A > B; A > D
HADS-A	9.30 (4.16)	7.12 (4.18)	9.32 (4.28)	6.16 (4.98)	4.92	.002	0.07	A > D; C > D

*BE* Binge eating; *BMI* Body Mass Index; *DDF* difficulty describing feelings; *DIF* difficulty identifying feelings; *EOT* externally orientated thinking; *HADS‐A* Hospital Anxiety and Depression Scale‐anxiety subscale; *HADS‐D* Hospital Anxiety and Depression Scale‐depression subscale; *MetS* Metabolic Syndrome

^a^Patients with MetS and BE,

^b^Patients with MetS and without BE

^c^Patients without MetS and with BE

^d^Patients without MetS and BE

### Between-variable associations

Multiple significant correlations in the moderate range (*r* = 0.30–0.50) were found among variables. Alexithymia was significantly associated with psychological distress and BE. In particular, the TAS-20 total and the DIF subscale correlated higher with HADS-D (*r* = 0.43 and *r* = 0.46, respectively) and at a lower level with HADS-A and BES scores (*r* = 0.30–0.35). The DDF subscale and BES also showed significant correlations with psychological distress within the same correlation range. The highest correlation coefficient occurred between age and the duration of overweight (*r* = 0.83) (see Table 3).

**Table 3:** Zero-order correlations between socio-demographic, clinical and psychological variables of the study sample

Variable	1	2	3	4	5	6	7	8	9	10
1. Age	–									
2. Duration of overweight	0.83***	–								
3. BMI	− 0.03	0.11	–							
4. BES	− 0.14*	− 0.02	0.15*	–						
5. HADS-A	− 0.02	0.04	0.08	0.33***	–					
6. HADS-D	− 0.02	0.03	0.08	0.35***	0.49***	–				
7. TAS Total score	0.17**	0.22***	0.09	0.30***	0.31***	0.43***	–			
8. DIF	0.11	0.17*	0.17**	0.32***	0.33***	0.46***	0.86***	–		
9. DDF	0.13*	0.18**	0.09	0.23***	0.32**	0.33***	0.82***	0.63***	–	
10. EOT	0.17**	0.18**	− 0.07	0.11	0.04	0.17**	0.63***	0.26***	0.34***	–

All value are standardized regression weights

*BES* Binge Eating Scale; *BMI* Body Mass Index; *DDF* difficulty describing feelings; *DIF* difficulty identifying feelings; *EOT* externally orientated thinking; *HADS‐A* Hospital Anxiety and Depression Scale‐anxiety subscale; *HADS‐D* Hospital Anxiety and Depression Scale‐depression subscale; *TAS-20* 20-item Toronto Alexithymia Scale

^*^p < 0.05; **p < 0.01; ***p < 0.001

### Structural equation modeling

**Fig. 1:**
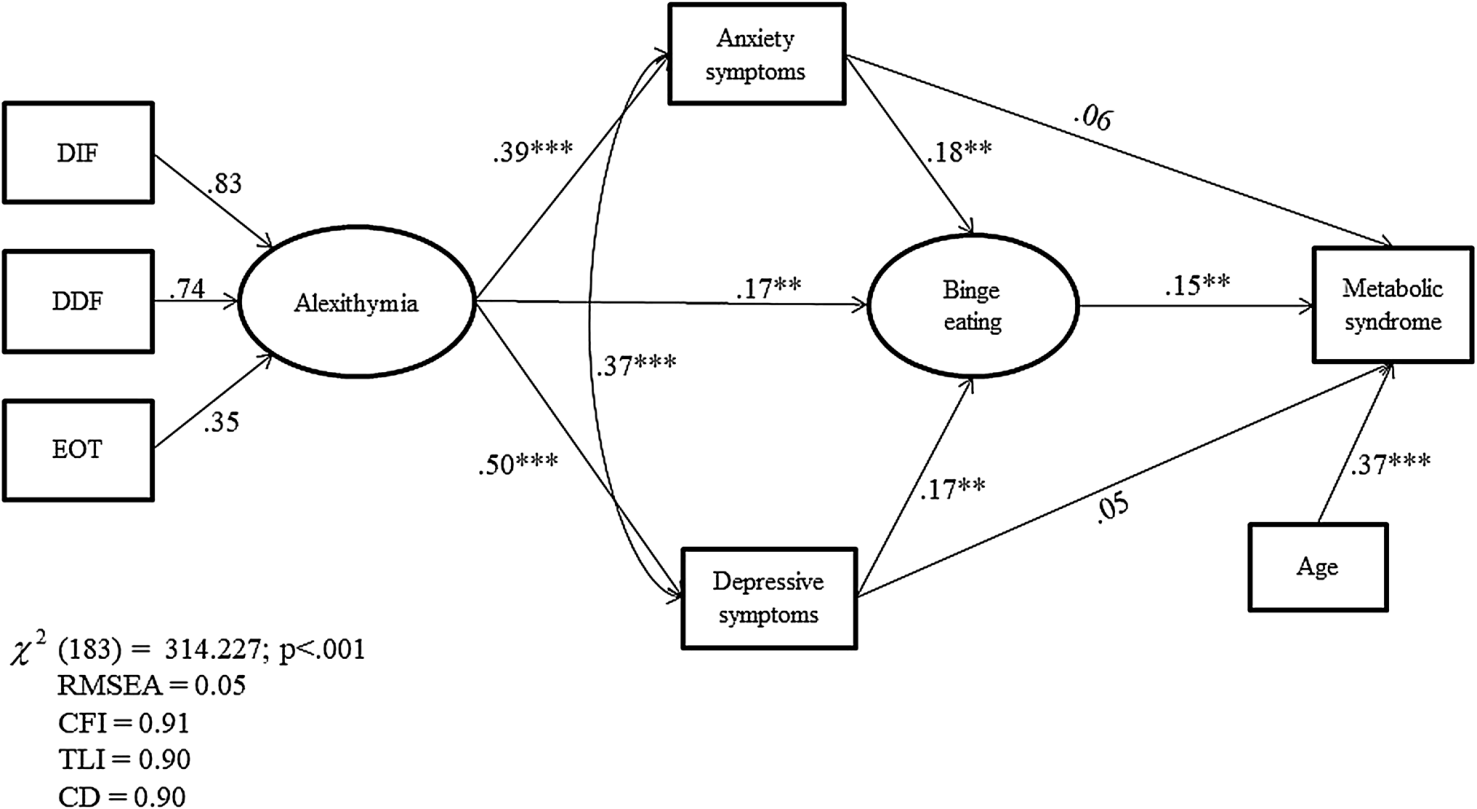
Structural equation modeling among alexithymia, binge eating, depression and anxiety symptoms, and MetS. *DDF* difficulty describing feelings; *DIF* difficulty identifying feelings; *EOT* externally orientated thinking. All value are standardized regression weights. *p < 0.05; **p < 0.01; ***p < 0.001

Figure 1 shows the path analysis and parameter estimates. All the observed variables were loaded on their corresponding latent constructs, supporting the validity of each latent construct, and standardized residuals were normally distributed. The parameter model estimates indicated that BE exerted a significant direct positive effect on MetS (*β* = 0.15), whereas there were no significant direct effects of HADS-A and HADS-D (*β* = 0.06 and *β*= 0.05, respectively). A significant direct effect of alexithymia was found on BE (*β* = 0.17), and depression (*β* = 0.50) and anxiety symptoms (*β* = 0.39). In addition, a significant direct effect of depression and anxiety symptoms (*β* = 0.17and *β* = 0.18, respectively) was found on BE. Finally, a significant direct effect of age as a covariate on MetS (*β* = 0.38), and a significant positive correlation between HADS-A and HADS-D were found (*β* = 0.37, *p* < 0.001). Put in more clinically bound words, results of SEM indicate that: *(a)* the higher the alexithymia level, the greater the increase of BE behaviors and psychological distress; *(b)* the higher the increase of psychological distress, the greater the increase of BE behaviors; and *(c)* the higher the BE behaviors, the greater the increase in MetS.

SEM showed positive and indirect effects of alexithymia (*β*^indirect^ = 0.10, *p* = 0.01) on MetS through the mediation of BE behaviors and depression and anxiety symptoms. In addition, a positive and indirect effect of depression (*β*^indirect^ = 0.02, *p* = 0.05) and anxiety symptoms (*β*^indirect^ = 0.03, *p* = 0.01) was found on MetS through the mediation of BE behaviors (see Table 4). Finally, the total effect of alexithymia (*β* = 0.11, *p* = 0.01), BE (*β* = 0.14, *p* = 0.01), and age (*β* = 0.37, *p* = 0.001) on MetS was significant, but there were no significant total effects of psychological distress on MetS. The values of multiple fit indices indicated that the proposed model provided a good fit of the data, *χ*^2^(183) = 314.227, *p* < 0.001, TLI = 0.90, CFI = 0.91, CD = 0.90, and RMSEA = 0.05.

**Table 4:** Indirect effects on MetS (*N* = 238)

	*β*	SE	*p*	*z*
HADS-A	0.03	0.01	0.01	2.35
HADS-D	0.02	0.01	0.05	2.02
TAS-20	0.10	0.02	0.01	2.98

*HADS-A* Hospital Anxiety and Depression Scale‐anxiety subscale; *HADS‐D* Hospital Anxiety and Depression Scale‐depression subscale; *TAS-20* 20-item Toronto Alexithymia Scale

In sum, within our sample of patients with obesity or overweight, the SEM analysis showed that the relationship between alexithymia and MetS was not linear and direct but partially mediated by BE and psychological distress. In addition, age was found as a major factor influencing MetS.

## Discussion

MetS is one of the major contributors to the epidemic of cardiovascular disorders in industrialized countries [7, 46, 47]. It should be noted that MetS is a heterogeneous label including hypertension, dyslipidemia, and hyperglycemia, which are all highly comorbid and correlated, although their pathophysiology does not necessarily overlap. One of the main utilities of the diagnostic label of MetS is to provide an opportunity to identify high-risk populations, such as individuals with obesity, and prevent the progression of some major causes of morbidity and mortality [48]. Several biomedical (e.g., insulin resistance, abdominal obesity, and increased activity of proinflammatory cytokines), lifestyle (e.g., smoking habits, lack of exercise, and disordered eating patterns), and psychiatric (e.g., mood, anxiety, schizophrenia, alcohol abuse, and personality disorders) risk factors for MetS have been identified [47, 48]. However, less is known about personality dimensions that may contribute to the development of MetS, particularly in high-risk populations, such as individuals with obesity or overweight. Alexithymia is considered a non-specific, trans-diagnostic construct associated with BE and obesity. The present study investigated whether alexithymia may constitute a potential risk factor for the development of MetS in outpatients with obesity or overweight, and whether a BE behavioral pattern and psychological distress may play a mediating role.

In our first hypothesis, we expected that patients with MetS would exhibit more features of alexithymia, BE, depression, and anxiety than non‐MetS obese patients. This hypothesis was fully confirmed. Patients with obesity or overweight and comorbid MetS had more alexithymic deficiencies (particularly difficulties in identifying and communicating their feelings), reported more behavioral patterns of BE, and were more psychologically distressed than those without MetS. These results are consistent with findings from previously published prospective studies on psychological factors and their co-occurrence with MetS and its components [28, 49]. For example, in a prospective population-based study, subjects with high psychological distress at baseline were more than twice as likely to develop MetS than those with low psychological distress, even after adjusting for sociodemographic variables, health behaviors, and C-reactive protein [50].

Consistent with the literature, our patients showed a close link between alexithymia and psychological symptoms (i.e., anxiety, depression, BE). A substantial body of evidence indicates that alexithymia and affective symptoms largely overlap, and that it is difficult to disentangle the causative role played by either one of these overlapping factors [31]. A number of longitudinal studies have shown that across a variety of clinical and non-clinical samples that alexithymic traits are relatively stable overtime. However, there can be some fluctuation in these trait alongside changes in anxiety and depression symptoms, further highlighting the intricate relationship between these variables [51, 52]. In our study, the association between alexithymia and duration of overweight suggests that there may be a vicious cycle between emotional dysregulation, overtime persistence of the clinical condition, and BE behavior. This is also in line with our second hypothesis that patients with MetS and BE would have higher alexithymic traits than all other patients. Our results showed that patients with MetS and BE had significantly higher alexithymia and DIF scores than those without MetS and BE. These results are consistent with findings that difficulties with emotional dysregulation may lead to compensatory overeating and, in turn, failed weight-loss attempts, which may further exacerbate difficulties in down-regulating emotional arousal, a pattern that has been repeatedly evidenced in alexithymic patients engaging in substance use and eating disorders [53]. Consistently, a number of longitudinal studies have demonstrated that personality traits characterized by inadequate stress control can increase the risk of MetS [49, 50].

In our third hypothesis, alexithymia was expected to indirectly affect MetS through the mediating role of BE behaviors and psychological distress. As expected, SEM analysis revealed that alexithymia affected MetS through the mediating role of BE, and depression and anxiety symptoms. This finding is in line with the results of our previous study that have emphasized the effect of alexithymic features on the severity of BE behaviors in patients with obesity [25]. The present study showed that alexithymia was linked to BE both directly and through the mediating effect of psychological distress. Several mechanisms may explain how this pathway can increase the risk of MetS. In a model proposed by Goldbacher et al. [54], psychological factors may contribute to the pathogenesis of MetS via physiological as well as behavioral mediating pathways. Several studies confirm that alexithymia is related to physical [55] and psychological distress [56], and directly associated with hyperarousal, sympathetic over-activity [57], and altered immune responses [58]. It may represent a personality marker of stress sensitivity or a vulnerability marker of stress, facilitating both unhealthy behaviors and inflammatory processes [57]. As inflammation is a recently purported physiological mechanism linking psychological distress to the development of MetS [59], alexithymia may be seen as a predisposing factor to illness not only through lifestyle factors but also through shared physiological factors.

### Limitations

There are several methodological limitations that require consideration when interpreting the findings of this study. First and foremost, a consecutive non‐probabilistic sampling method was used. Non-probabilistic recruitment can result in a biased sample that is not fully representative of the wider population. A probabilistic sampling procedure should, therefore, be utilized in future studies. Second, the cross‐sectional nature of the current study does not enable us to explore causal relationships between MetS, BE, and alexithymia. Prospective longitudinal studies should be used in the future to explore the timing and sequence of BE onset and various metabolic disruptions across the lifespan to tease apart these complex relationships. Third, alexithymia, psychological distress, and BE were only assessed using self-report scales. Self-reported scales require a considerable amount of personal insight and may be influenced by subjective biases and social desirability effects. Nonetheless, evidence indicates that the self-report TAS-20 is a valid and sound measure of alexithymia [60]. The integration of the TAS-20 within a multimethod assessment framework would be preferred, although more difficult in clinical settings [61]. Fourth, a number of potentially relevant factors were not controlled for, such as an objective measure or document of past psychopathology, lifetime and current psychiatric conditions, quality of life, sleep quality, social support, medical comorbidity, physical activity, dietary habits, and inflammation activity. Future investigations should take into account those factors as relevant moderating and mediating variables whose change overtime may influence the association of alexithymia and BE with MetS. Fifth, our patients were recruited in a secondary care center and seeking treatment for their clinical condition. Individuals with obesity or overweight actively seeking medical, dietary, and psychological help for their weight problem and eating behavior are likely to be more motivated to make behavioral changes and more aware of their psychological problems. Follow-up studies should investigate whether the role of alexithymia and BE is different in patients with obesity seeking treatment who completed the intervention program and those who are non-treatment seeking or dropped out.

### Clinical implications and conclusions

With these limitations in mind, the present findings indicate that alexithymia, particularly the facets of difficulty identifying and describing feelings, and BE have a relevant role in explaining the development of MetS in individuals with obesity or overweight, which may have important clinical implications. Clinicians should consider binge eating behavioral patterns and alexithymic characteristics in the clinical management of patients with MetS because these psychological aspects may influence the clinical presentation of the overall syndrome or its separate components. In addition, both alexithymia and BE can be modified through specific psychological interventions as well as integrated multicomponent treatment programs [62]. Screening for alexithymia and BE may, therefore, help in planning individualized, tailored treatment for improving MetS and its consequences on health.

In conclusion, we aimed to further explore the complex association between alexithymia and MetS by evaluating the mediating role of binge eating behavior and psychological distress. Our findings suggest that MetS is associated with distinct emotional and behavioral characteristics that require appropriate attention in the clinical management of patients.

## What is already known on this subject?

Overweight, obesity, and metabolic syndrome are an increasing global health challenge. Alexithymia has previously been associated with unhealthy behaviors and chronic medical conditions. Binge eating has not been considered as a co-variable in previous studies exploring the relationship of alexithymia with MetS. In this study, we aimed to explore the association between alexithymia and MetS by including maladaptive eating behavior and psychological distress as mediating factors.

## What this study adds?

Binge eating and psychological distress mediate the relationship between alexithymia and MetS. Our findings suggest that MetS is associated with distinct emotional and behavioral characteristics that require appropriate attention in the clinical management of patients.

## Data Availability

The datasets generated during and/or analyzed during the current study are available from the corresponding author on reasonable request.
